# Non-Canonical Cell Death Induced by p53

**DOI:** 10.3390/ijms17122068

**Published:** 2016-12-09

**Authors:** Atul Ranjan, Tomoo Iwakuma

**Affiliations:** Department of Cancer Biology, University of Kansas Medical Center, Kansas City, KS 66160, USA; aranjan@kumc.edu

**Keywords:** apoptosis, caspase-independent apoptosis (CIA), ferroptosis, necroptosis, autophagy, mitotic catastrophe, paraptosis, pyroptosis, efferocytosis

## Abstract

Programmed cell death is a vital biological process for multicellular organisms to maintain cellular homeostasis, which is regulated in a complex manner. Over the past several years, apart from apoptosis, which is the principal mechanism of caspase-dependent cell death, research on non-apoptotic forms of programmed cell death has gained momentum. p53 is a well characterized tumor suppressor that controls cell proliferation and apoptosis and has also been linked to non-apoptotic, non-canonical cell death mechanisms. p53 impacts these non-canonical forms of cell death through transcriptional regulation of its downstream targets, as well as direct interactions with key players involved in these mechanisms, in a cell type- or tissue context-dependent manner. In this review article, we summarize and discuss the involvement of p53 in several non-canonical modes of cell death, including caspase-independent apoptosis (CIA), ferroptosis, necroptosis, autophagic cell death, mitotic catastrophe, paraptosis, and pyroptosis, as well as its role in efferocytosis which is the process of clearing dead or dying cells.

## 1. Introduction

Cell death was first reported by Carl Vogt in 1842 [1]. It is an irreversible phenomenon and involves complex molecular factors or pathways to maintain cellular homeostasis and control diseases in multicellular organisms [2]. Apoptosis is the best-characterized program of cell death that is crucial for maintenance of tissue homeostasis, embryonic development, and immune regulation [3,4]. Its biological features are characterized by cell size reduction, membrane blebbing, chromatin condensation, DNA fragmentation, and formation of apoptotic bodies which are finally engulfed by phagocytes [5]. Apoptosis can be triggered by a wide variety of stimuli, such as DNA damage (e.g., irradiation, chemotherapy drugs), hormones (e.g., corticosteroids in thymocytes), and activation of death receptors (e.g., CD95/APO-1/FAS; tumor necrosis factor receptor: TNFR) [6]. Apoptosis can be categorized into (a) the extrinsic (death receptor) pathway mediated mainly by FAS and TNFR [7,8]; and (b) the non-receptor-mediated intrinsic pathway primarily involving mitochondrial-initiated events [9]. Stimuli such as cytokines, hormones, and pathogens (*Mycobacterium* and *Chlamydia pneumoniae*) are responsible for the extrinsic pathway, whereas stimuli such as oxidative stress (reactive oxygen species, ROS), irradiation, chemotherapy drugs, and endoplasmic reticulum stress induce the intrinsic pathway [10,11,12,13,14]. In the extrinsic pathway, binding of ligands to their respective receptors induces oligomerization and activation of caspase-8, which in turn activates caspase-3, leading to apoptosis [15]. Intrinsic stimuli trigger mitochondrial outer membrane permeabilization (MOMP) mediated by the B-cell lymphoma 2 (Bcl-2) family of proteins (pro-apoptotic Bcl-2-associated X: BAX; Bcl-2-associated death promoter: BAD; Bcl-2-antagonist/killer: Bak or anti-apoptotic Bcl-2; B-cell lymphoma-extra large: Bcl-xL). This causes the release of cytochrome c (cyt c) from mitochondria to the cytoplasm that binds with apoptotic protease activating factor 1 (Apaf-1), leading to activation of caspase-9 and subsequently caspase-3 [6]. Thus, both pathways involve activation of caspases which belong to a family of cysteine protease enzymes [8,16,17]. Mutations in apoptosis-related genes abrogate the cell death process, which has become one of the major challenges in treating cancer and other diseases.

Different types of non-apoptotic cell death have been identified through recent studies in various species including *Caenorhabditis elegans*, *Drosophila melanogaster*, humans, and other multicellular organisms [18,19]. These non-canonical cell death mechanisms include caspase-independent apoptosis (CIA), ferroptosis, necrosis, autophagy, mitotic catastrophe, paraptosis, and pyroptosis (Table 1). They are different in the causative factors, morphological features, and proteins or pathways involved [18]. Many of these non-apoptotic non-canonical cell death pathways are independent of caspases that are used for apoptosis induction [18,19]. These cell death machineries could be used as secondary modes of cell death when apoptosis fails [20].

p53, the most frequently mutated tumor suppressor in human cancers, functions as a tumor suppressor by transcriptionally regulating numerous downstream target genes involved in cell cycle progression and cell death as a transcription factor [21,22]. p53 is stabilized and activated by external and internal stress signals [23]. Activated p53 induces apoptosis by transactivating pro-apoptotic genes (e.g., *BAX*, *BAD*, *Bak*) [24]. p53 also directly binds to anti-apoptotic mitochondrial proteins (e.g., Bcl-2, Bcl-xL) and efficiently induces apoptosis [25]. Apoptosis mediated by p53 is crucial for tumor suppression, radiosensitivity, and chemosensitivity [26]. Recent evidence has suggested that p53 is also directly or indirectly involved in several non-canonical cell death mechanisms, which will be discussed in this review article.

## 2. Caspase-Independent Apoptosis (CIA)

**Figure 1:**
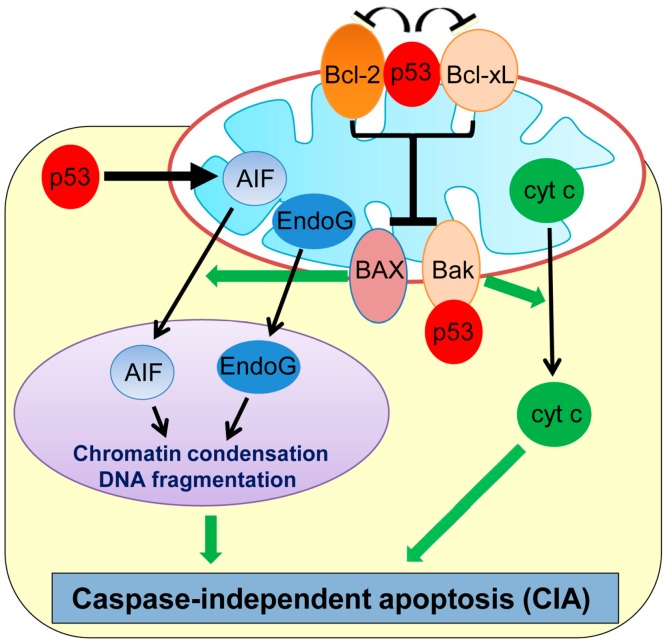
Schematic representation of caspase-independent apoptosis (CIA) and its regulation by p53. p53 transcriptionally upregulates AIF, binds and inhibits Bcl-2 and Bcl-xL, and also binds with Bak to induce CIA. AIF: Apoptosis-inducing factor; cyt c: cytochrome c; EndoG: endonuclease G.

Recent studies have revealed that cell death can also occur in a caspase-independent manner, namely caspase-independent apoptosis (CIA). Although CIA still requires some of the upstream signaling pathways and MOMP similar to apoptosis, it is morphologically distinct from and is relatively a slower process than apoptosis (Figure 1) [27,28]. Many genotoxic stresses trigger MOMP and cause CIA even in the absence of caspases. Interestingly, plumbagin, a phytochemical compound used in the Indian system of medicine as an anti-inflammatory and anti-tumor compound, is shown to induce CIA in human colon cancer HCT116 and breast cancer MCF7 cell lines deficient in BAX [29,30]. The chemotherapeutic drug, paclitaxel, also induces CIA in ovarian carcinoma cells [31] and non-small cell lung cancer cells [32].

Apoptosis-inducing factor (AIF) and endonuclease G (EndoG), two main factors released from mitochondria, play primary roles in CIA (Figure 1) [33,34,35]. AIF is a ubiquitous mitochondrial oxidoreductase and mainly functions as a scavenger of reactive oxygen species (ROS) [36]. However, upon apoptotic stimuli, AIF is released from the mitochondria to the nucleus, which triggers large-scale DNA fragmentation and nuclear chromatin condensation, leading to CIA [37]. A flavin adenine dinucleotide (FAD)-binding domain in the N-terminal region of AIF is required for inducing CIA [38]. Inhibition of AIF translocation to the cytoplasm or treatment with AIF-neutralizing antibodies is shown to inhibit CIA induced by DNA damage or pneumococcus infection in fibroblasts and neurons [33,39,40]. EndoG is also shown to translocate from mitochondria to the nucleus upon genotoxic stress and is capable of inducing DNA fragmentation and CIA in mammalian cells, including mouse embryonic fibroblasts (MEFs) deficient in the *DNA fragmentation factor-45 (DFF45/DFFA)* gene which is a crucial player for caspase-dependent DNA fragmentation [34].

In neuronal cells, p53 is shown to induce delayed-onset CIA via AIF release from mitochondria [39]. Direct binding of mitochondrial p53 with Bcl-xL and Bcl-2 results in neutralization of their inhibitory effects on pro-apoptotic BAX and Bak which also play roles in CIA (Figure 1) [30,41,42]. Cregan et al. [39] show that BAX regulates the mitochondrial release of AIF and induces CIA. In addition, phosphorylated p53 directly binds to Bak which causes oligomerization of Bak and cyt c release from mitochondria, leading to CIA [30,41]. Additionally, p53 transcriptionally represses Bcl-2 and also upregulates BAX [43,44], which could contribute to p53-mediated CIA. Moreover, p53 can directly upregulate transcription of the *AIF* gene and sensitize human non-small cell lung carcinoma cells (H1299) to CIA [45]. Thus, p53 induces CIA by the transcriptional regulation of and physical binding to CIA mediators.

## 3. Ferroptosis

**Figure 2:**
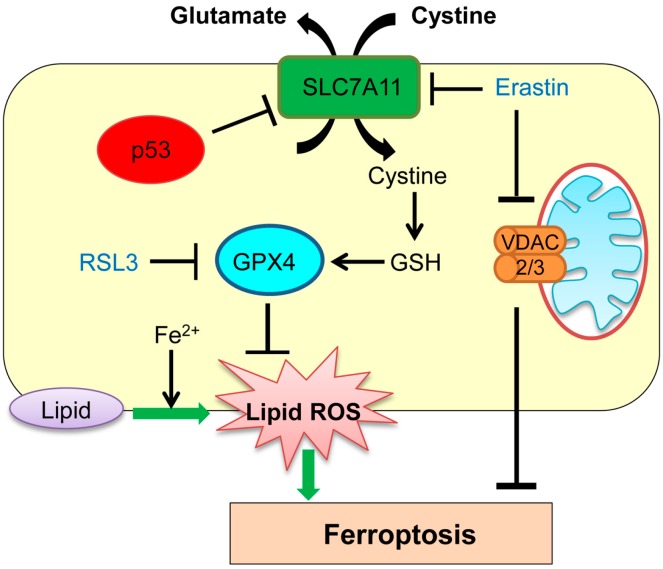
Role of p53 in ferroptosis. p53 transcriptionally represses solute carrier family 7 member 11 (SLC7A11), sensitizing cells to ferroptosis. GSH: glutathione; GPX4: glutathione peroxidase 4; ROS: reactive oxygen species; RSL3: Ras selective lethal 3; VDAC: voltage-dependent anion channels.

Ferroptosis has been previously detected in the brain in cases of exposure to high levels of glutamate, and in the kidney and heart with ischemia–reperfusion injury [46,47,48,49,50]. Ferroptosis represents intracellular iron-dependent cell death and is independent of caspases, BAX, Bak, autophagy inhibition, and Ca^2+^ influx [46,51,52,53,54,55]. Ferroptosis occurs through accumulation of toxic lipid ROS induced by iron molecule via inhibition of cystine import, depletion of glutathione biosynthesis, and inhibition of the glutathione-dependent antioxidant enzyme GPX4 (glutathione peroxidase 4; Figure 2). It can also be induced by treatment of cells with small molecules, erastin and RSL3 (Ras selective lethal 3; Figure 2) [46,53]. Iron chelation effectively inhibits the erastin- and RSL3-induced ferroptosis [46].

Studies have shown that cancer cells with mutations in the RAS (rat sarcoma)-RAF (rapidly accelerated fibrosarcoma)-MEK (mitogen-activated protein kinase/extracellular signal–regulated kinase kinase) pathways can be selectively targeted by activation of ferroptosis [55]. In line with this study, ferroptosis is preferentially induced in a Harvey (H)-RAS^G12V^-expressing human fibroblast BJ cell line by treatment with erastin and RSL3, as compared with BJ cells without HRAS^G12V^ [56]. However, the exact mechanism of the observed synthetic lethality remains unclear. Intriguingly, mechanisms of ferroptosis induction by erastin and RSL3 are different. Erastin interferes with the cellular metabolism by binding to voltage-dependent anion channels 2 and 3 (VDAC2/3), resulting in mitochondrial dysfunction and subsequent induction of ferroptosis [55]. Erastin also induces ferroptosis by selectively inhibiting an amino acid antiporter solute carrier family 7 member 11 (SLC7A11; also known as system X_c_^−^ or xCT) that mediates the exchange of extracellular l-cystine with intracellular l-glutamate across the cell membrane (Figure 2) [46]. On the other hand, RSL3 binds to and inactivates the peroxidase activity of GPX4, thus inducing ferroptosis (Figure 2) [53].

Recent studies have suggested that ferroptosis regulated by p53 plays a crucial role in tumor suppression. Jiang et al. [57] show that p53 represses transcription of the *SLC7A11* gene through a p53-responsive element in the 5′ flanking region. Inhibition of cystine uptake via reduced SLC7A11 levels by p53 sensitizes cells to ferroptosis (Figure 2). They also show that an acetylation-defective p53 mutant (3 lysine (K) to arginine (R): K117R, K161R, and K162R) lacking the abilities of inducing cell cycle arrest, senescence, and apoptosis, can still reduce SLC7A11 levels and hence maintain the ability to induce ferroptosis [57]. These results strongly suggest that ferroptosis through p53 occurs in a manner independent of other p53-regulated cellular activities [57,58,59].

## 4. Necroptosis

Necrosis was previously thought as accidental, non-programmed, and unregulated form of cell death, resulting from exposure of cells to extreme physicochemical conditions [60]. During necrosis, cell membranes become permeable, which is followed by appearance of numerous cytoplasmic vacuoles filled with cellular remnants and rupture of the plasma membrane, hence causing inflammation [60,61]. Also, moderate chromatin condensation, chromatin clumping, and random degradation of DNA are detected in the nucleus [60,61,62].

**Figure 3:**
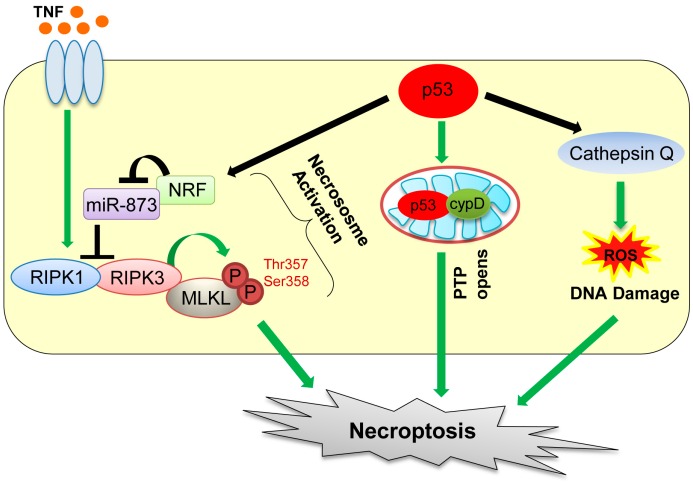
Mechanisms of necroptosis mediated by p53. p53 induces necroptosis by transactivating necrosis-related factor (NRF) and cathepsin Q and also directly binding with cyclophilin D (cypD). TNF: tumor necrosis factor; MLKL: mixed lineage kinase domain-like protein; miR: microRNA; PTP: permeability transition pore; RIPK: receptor-interacting serine/threonine protein kinase.

Recent studies have revealed that necrotic cell death can also be caused by defined molecular pathways. The regulated form of necrotic cell death is referred to as “necroptosis” [63,64]. Death receptor ligands, including the tumor necrosis factor (TNF) and FAS ligand, are the two best known inducers of necroptosis [65,66]. Necroptosis is dependent on activities of two related kinases, receptor-interacting serine/threonine kinase protein 1 (RIPK1) and RIPK3 (Figure 3) [64,67,68]. Specifically, necroptosis is initiated by TNF binding to its receptor, which promotes the interaction between RIPK1 and RIPK3, followed by activation of these kinases and formation of the necrosome complex with mixed lineage kinase domain-like protein (MLKL) (Figure 3) [69]. Phosphorylation of MLKL by RIPK3 at threonine 357 and serine 358 residues enhances its oligomerization, which promotes translocation of MLKL oligomers from the cytosol to the plasma membranes to disrupt membrane integrity, causing necroptosis [70,71]. However, there is a report showing that TNF can also activate RIPK3 to induce necroptosis in MEFs, even in the absence of RIPK1 [72].

p53 also plays a role in oxidative stress-induced necroptosis. Tu et al. [73] show that etoposide can induce necroptosis in *BAX/Bak* double knockout MEFs. This is caused by cooperation of DNA damage-induced ROS with increase in cathepsin Q levels induced by p53 (Figure 3) [73]. Also, during oxidative stress, p53 is accumulated in the mitochondrial matrix, which enhances opening of mitochondrial permeability transition pore (PTP) via direct binding of p53 with a PTP regulator cyclophilin D (cypD), leading to mitochondrial swelling and induction of necroptosis (Figure 3) [74]. Recently, Wang et al. [75] showed that upon ischemia/reperfusion injury, p53 transcriptionally upregulates a long-noncoding RNA (lncRNA), named necrosis-related factor (NRF; Figure 3). Since necrosis-related factor (NRF) functions as an endogenous sponge RNA to repress microRNA-873 (miR-873) expression and miR-873 suppresses translation of RIPK1/RIPK3, NRF upregulation by p53 leads to decrease in miR-873 and increase in RIPK1/RIPK3 levels, leading to induction of necroptosis [75]. Thus, direct and indirect contributions of p53 to necroptosis have increasingly been recognized and likely have clinical implications in stroke pathology (transient ischemia–reperfusion injury of the brain) [74]. Indeed, inhibition of necroptosis by RIPK1 or mixed lineage kinase domain-like protein (MLKL) inhibitors is shown to be beneficial for ischemia–reperfusion injury in animal models [63]. However, this remains to be tested in human clinical trials.

## 5. Autophagic Cell Death

**Figure 4:**
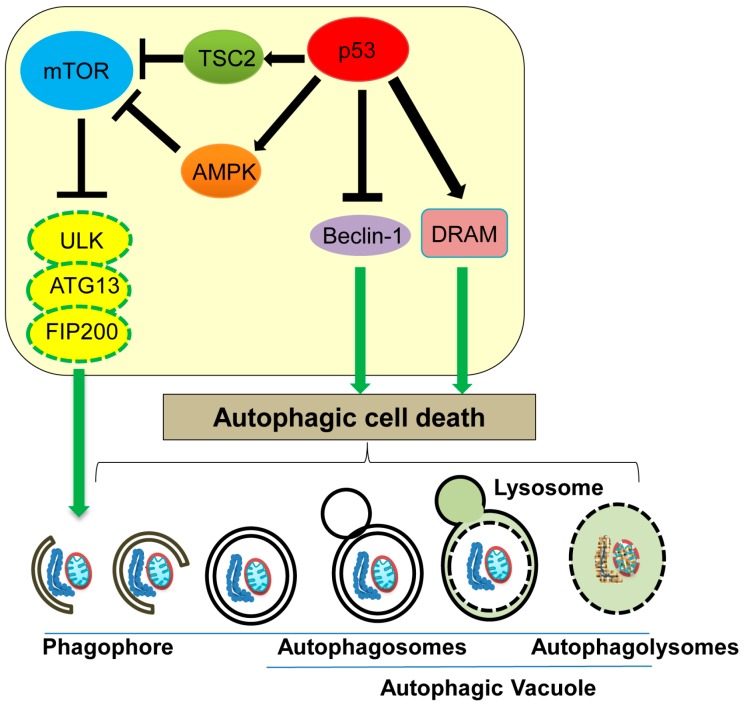
Autophagic cell death and its regulation by p53. Nuclear p53 induces autophagic cell death by transcriptionally upregulating tuberous sclerosis (TSC2), AMP-activated protein kinase (AMPK), and damage-regulated autophagy modulator (DRAM), whereas cytoplasmic p53 inhibits autophagic cell death by inducing Beclin-1 degradation. mTOR: mammalian target of rapamycin.

Autophagy can control cell survival or cell death depending on the cellular context [2]. Autophagic cell death is a non-apoptotic, non-necrotic cell death which results from the process of autophagy [2]. Autophagy is a regulated catabolic lysosomal-dependent process, which facilitates cells to eliminate damaged or non-functional cellular components (mitochondria, endoplasmic reticulum, peroxisomes), misfolded proteins, and pathogens, in order to maintain cellular homeostasis [18,76,77]. During autophagy, cytoplasmic contents including organelles are sequestered by unique isolated membranes called phagophores to form autophagosomes. Later, the mature autophagosome merge with lysosomes to form autophagolysomes which degrade the engulfed materials (Figure 4).

Autophagy is conserved in various organisms and is mostly triggered by nutrient starvation. The endocrine system also regulates autophagy; specifically, insulin suppresses autophagy in the liver, while glucagon enhances it [78].

ATG (AuTophaGy-related) proteins and Beclin-1 are well characterized mediators of the autophagic process [79]. ATG13, one of the ATG proteins, forms a complex together with unc-51 like autophagy activating kinase 1 (ULK1) and focal adhesion kinase interacting protein of 200 kD (FIP200), which is involved in phagophore formation and is essential for nutrient starvation-induced autophagy [80]. mTOR (mammalian target of rapamycin), a protein kinase that controls cell cycle progression and protein translation, can phosphorylate and inhibit activities of ATG13 and ULK1, leading to inhibition of phagophore formation and hence autophagy (Figure 4) [80]. In response to nutrient starvation or rapamycin treatment, mammalian target of rapamycin (mTOR) activity is inhibited, which activates the ATG13-ULK-FIP200 complex, leading to induction of autophagy [81]. Thus, mTOR functions as a master regulator of autophagy upon nutrient deprivation [82]. Additionally, Beclin-1 is shown to induce the autophagic cell death by promoting autophagosome formation, even in the absence of apoptosis using cells lacking BAX and Bak (Figure 4) [83]. Interestingly, a decrease in Beclin-1 levels is observed in human breast and liver cancer [84,85,86].

Accumulating evidence indicates that p53 can both promote and inhibit the autophagic process, depending on its subcellular localization; nuclear p53 promotes autophagy, while cytoplasmic p53 inhibits it [87]. Nuclear p53 transactivates TSC2 (tuberous sclerosis) and AMPK (AMP-activated protein kinase), both of which downregulate mTOR activity, thus indirectly promoting autophagy (Figure 4) [88,89]. Sestrins 1 and 2, activators of AMPK, are also upregulated by stress-induced p53 [90]. DRAM (damage-regulated autophagy modulator) is a well-studied stress-induced activator of autophagy and is transcriptionally upregulated by p53 (Figure 4) [91,92,93]. Additionally, several p53-regulated apoptosis players, including p53 upregulated modulator of apoptosis (PUMA), BAX, BCL2 interacting protein 3 (Bnip3), and BAD, are also implicated in promoting autophagy [94,95,96,97]. Thus, nuclear p53 induces the autophagic process by transactivating its downstream target genes [87,88]. On the other hand, cytoplasmic p53 is shown to bind with Beclin-1 and promote its ubiquitination and degradation, therefore inhibiting the process of autophagy [98]. Tasdemir et al. [99] find that inhibition of p53 can induce autophagy in nematodes and mammalian cells, and cytoplasmic p53 is mainly responsible for this autophagy inhibition. Thus, cytoplasmic p53 appears to inhibit autophagy. Further studies are required to investigate the context-dependent role of p53 in either promoting or inhibiting autophagy.

Many diseases have been linked with impaired autophagy. These include static encephalopathy of childhood with neurodegeneration in adulthood (SENDA) [100], Vici syndrome [101], hereditary spastic paraplegia (HSP) [102], lysosomal storage disorders [103,104], and cancers [105,106]. Autophagy inhibitors, such as chloroquine and hydroxychloroquine, are currently in clinical trials for multiple types of cancer [106]. On the other hand, autophagy inducers including trehalose, carbamazepine, and lithium carbonate are in clinical trials to treat vascular aging, α1-antitrypsin deficiency liver cirrhosis, and amyotrophic lateral sclerosis, respectively [106].

## 6. Mitotic Catastrophe

**Figure 5:**
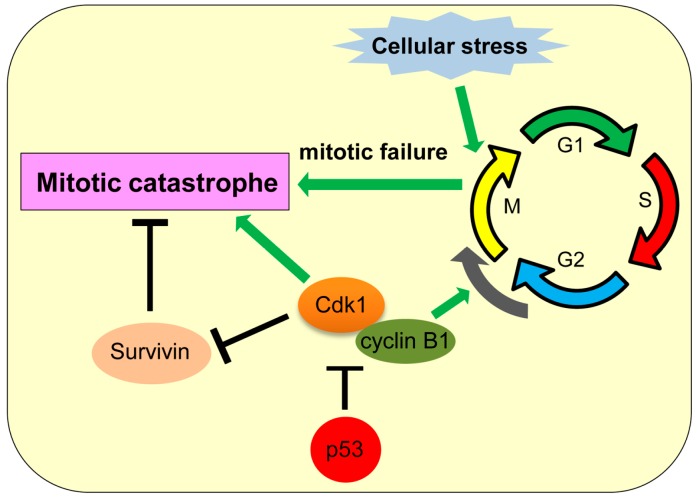
Mitotic catastrophe and its association with p53. p53 inhibits transcription of cyclin-dependent kinase 1 (cdk1) and cyclin B1 to inhibit mitotic catastrophe.

Mitotic catastrophe is a type of cell death caused by failed or incomplete mitosis, which results in chromosomal breaks and poor karyokinesis with multinucleation and micronucleation [107]. Mitotic catastrophe is triggered by DNA damage, anomaly in mitotic (M) phase and spindle checkpoint of the cell cycle, microtubular poisons, tetraploidy, and hyperthermia [108]. Mitotic catastrophe was first described in yeast as a result of abnormal chromosome segregation due to overexpression of cyclin-dependent kinase 1 (cdk1 or cdc2) [109,110,111]. During mitotic progression, the cdk1/cyclin B1 complex promotes progression from gap 2 (G2) to M phase of the cell cycle and also plays roles in chromatin condensation, nuclear membrane breakdown, and microtubule reorganization during mitosis [112,113,114]. At the end of metaphase, the anaphase-promoting complex (APC) induces degradation of cyclin B and securin, allowing anaphase transition [112,115]. Prolonged inhibition of APC can lead to prolonged cdk1 activation which promotes mitotic catastrophe (Figure 5) [116]. Also, treatment of colon cancer cells with 5-fluorouracil and doxorubicin is shown to increase cyclin B1 levels and induce mitotic catastrophe [117,118]. Survivin, a protein required for mitotic progression and apoptosis inhibition, is frequently upregulated in cancers and plays a role in inhibiting mitotic catastrophe [119]. Phosphorylation of survivin by cdk1 inhibits its function, hence promoting mitotic catastrophe (Figure 5) [120]. Thus, proper function of cell cycle checkpoint regulators is vital for avoiding mitotic catastrophe. Indeed, inhibition of G2M checkpoint regulators, such as ATM (ataxia telangiectasia mutated), ATR (ataxia telangiectasia mutated and Rad3 related), Chk1 (checkpoint kinase 1), Chk2 (checkpoint kinase 2), and 14-3-3-σ, is also shown to induce mitotic catastrophe [107,118,121,122].

The inhibitory role of p53 in mitotic catastrophe has been reported. p53 inhibits transcription of cdk1 and cyclin B1, hence inhibiting mitotic catastrophe (Figure 5) [123,124]. p53 also upregulates expression of cdk1 inhibitors, including 14-3-3σ, p21, and GADD45 (growth arrest and DNA damage-inducible 45) [125,126,127,128]. Thus, p53 inhibits mitotic catastrophe in multiple ways. It is also shown that mitotic catastrophe occurs predominantly in p53-null cells due to aneuploidy and genomic instability [129]. However, there is a report showing that p53 can transcriptionally repress survivin [130]. Future studies need to clarify whether the inhibitory effect of p53 on survivin levels could promote mitotic catastrophe in some cellular contexts.

Mitotic catastrophe induced by anti-mitotic agents has been implicated in cancer therapy. Microtubule stabilizers, (taxanes: paclitaxel and docetaxel), are used in the treatment of breast cancer, ovarian cancer, non-small-cell lung cancer, and prostate cancer, while microtubule destabilizers, (vinca alkaloids: vinblastine and vincristine), are used to treat hematological malignancies [131,132,133]. Thus, induction of mitotic catastrophe is an efficient strategy for cancer therapy.

## 7. Paraptosis

**Figure 6:**
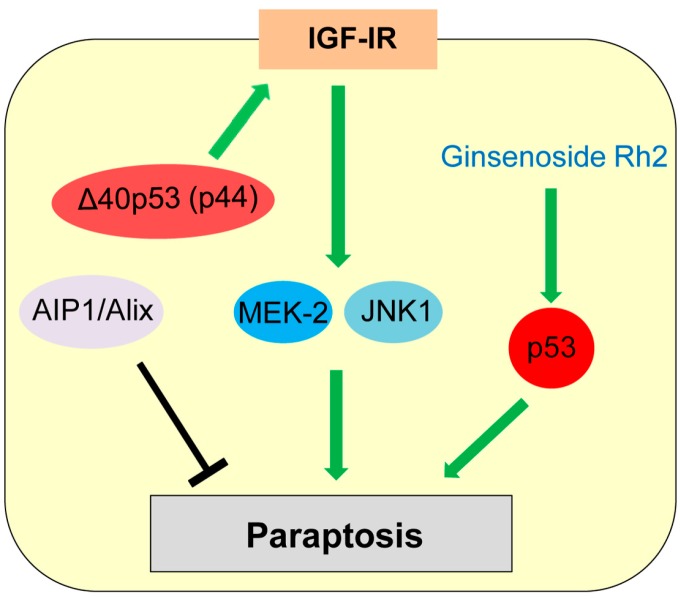
Role of p53 in paraptosis. Δ40p53 (p44) increases insulin-like growth factor I receptor (IGF-IR) levels, which induces paraptosis dependent on mitogen-activated protein kinase (MAPK) member proteins (MEK-2, JNK1).

Paraptosis was first reported by Sperandio et al. in the year 2000 as a type of programmed cell death displaying swelling of mitochondria and/or endoplasmic reticulum (ER) and cytoplasmic vacuolization [2,134]. Paraptosis is observed during development and neurodegeneration and can be induced by expression of insulin-like growth factor I receptor (IGF-IR) [2,134,135]. Paraptosis is also triggered in cancer cells treated with various natural and synthetic anti-cancer agents [136]. Sugimori et al. [137] report that benfotiamine, a drug used to treat diabetic neuropathy, induces paraptosis in acute myeloid leukemia (AML) cells. Unlike apoptosis, paraptosis is not affected by caspase inhibitors or overexpression of Bcl-2-like anti-apoptotic proteins [135]. Later, Sperandio et al. [135] also found that paraptosis induced by IGF-IR is mainly dependent on mitogen-activated protein kinase (MAPK) family members (Mitogen-activated protein kinase kinase 2: MEK-2; c-Jun N-terminal protein kinase 1: JNK1) and can be inhibited by AIP1 (ALG-2-interacting protein, also known as Alix, ALG-2 interacting protein-X; Figure 6).

Only a few studies show potential association of p53 with paraptosis. Treatment with ginsenoside Rh2, one of the active principles of Panax ginseng root, induces cell death by both apoptosis and paraptosis, in a p53-dependent manner [138]. Pehar et al. [139] report that mice expressing Δ40p53 (p44), a N-terminal region lacking isoform of p53, show neurodegeneration and premature death caused by paraptosis and autophagic cell death, due to elevated hyperactive IGF-IR signaling (Figure 6). However, p53 is also reported to inhibit the IGF-IR signaling by decreasing the *IGF-IR* promoter activity and upregulating IGF-BP3 expression [140,141]. Future investigations are required for clarifying the role of p53 in paraptosis induction.

## 8. Pyroptosis

**Figure 7:**
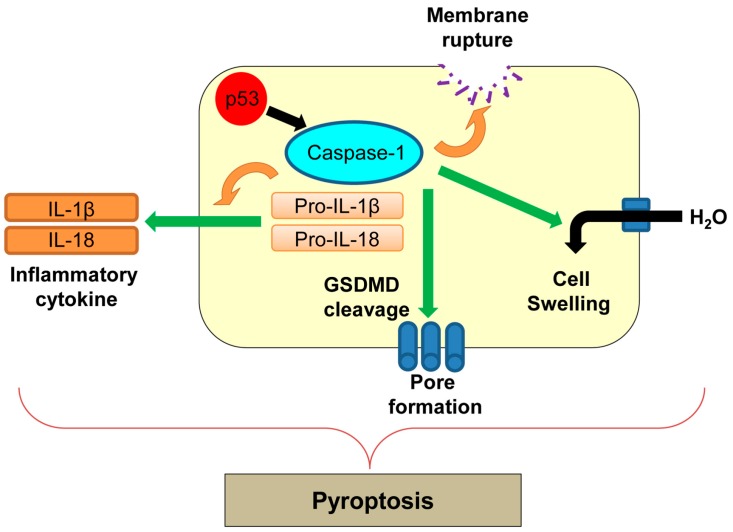
Caspase-1-mediated pyroptosis. p53 transcriptionally upregulates caspase-1, which could activate pyroptosis. GSDMD: gasdermin D; IL: interleukin.

Pyroptosis was first reported in macrophages infected with *Shigella flexneri* [24] and later in those infected with *Salmonella typhimurium* [142]. Pyroptosis is an inflammatory form of regulated cell death which is triggered by microbial infection, heart attack, stroke, and cancer progression [142]. Pyroptosis is morphologically and mechanistically different from other types of cell death [142]. During pyroptosis, there is rapid plasma membrane rupture and release of proinflammatory contents from infected cells [143]. The process of pyroptosis is mainly mediated by caspase-1 (Figure 7) [143]. Caspase-1 is an inflammatory caspase and is activated by various inflammatory molecules within the inflammasome, a multiprotein complex consisting of sensor and effector molecules for pathogens [144]. Active caspase-1 cleaves pro-inflammatory cytokines, interleukin-1β (IL-1β) and IL-18, and induces secretion of these cytokines (Figure 7) [145]. Activation of caspase-1 also cleaves a key component of inflammasomes, gasdermin D (GSDMD), which oligomerizes in membranes to accelerate non-selective pore formation on the plasma membrane [146]. This causes increased water influx, cell swelling, and membrane rupture, leading to cell lysis during pyroptosis [147].

There is no direct evidence showing correlation of p53 with pyroptosis. In addition, Hilbi et al. [148] show that *Shigella* infection-induced caspase-1-mediated cell death in macrophages is p53-independent. However, p53 transcriptionally upregulates caspase-1 [149]. Further studies are required to determine whether regulation of caspase-1 by p53 could induce pyroptosis in different cellular contexts. 

## 9. Efferocytosis

Recently, efferocytosis has been described as the process by which phagocytes engulf and digest dead or dying cells to form a fluid-filled vesicle containing dead cells, called efferosome [150,151,152]. Efferocytosis ensures the clearance of dying cells and protect neighboring tissue from the harmful and toxic effects of enzymes and other intracellular components released from the dead or dying cells [152]. Professional phagocytes recognize externalized negatively charged glycerophospholipid phosphatidylserine (PS) in dying cells through their receptors (e.g., the TIM (T cell/transmembrane, immunoglobulin, and mucin) and TAM (Tyro3-, Axl-, and Mer-tyrosine kinase) families of PS receptors, macrophage scavenger receptor CD36, and integrins), which triggers the engulfment of dying cells [150,153]. In *CD36* knockout mice, reduced efferocytosis is observed in lungs following bleomycin treatment [154,155]. miR-34a, a tumor suppressor transcriptionally induced by p53, is shown to negatively regulate efferocytosis by tissue macrophages, through reduction in the expression of an apoptotic cell-recognizing receptor tyrosine kinase Axl and NAD-dependent deacetylase sirtuin-1 [155,156]. Interestingly, efferocytosis downregulates the miR-34a expression, which in turn increases efferocytosis, thus forming a positive feedback loop [155]. In support of this study, p53-deficient macrophages are shown to have defective efferocytosis [157]. Thus, p53 is also involved in clearing dead or dying cells following cell death.

## 10. Conclusions and Discussion

Accumulating evidence indicates that mammalian organisms exhibit various types of regulated cell death apart from apoptosis, each of which has a different mechanism. In this review article, we have discussed seven different cell death mechanisms, as well as efferocytosis, which is the process of clearing dead or dying cells (Table 1). However, p53 might also be involved in other types of cell death, such as keratinization-associated cell death (cornification), anoikis (anchorage-dependent cell death), NETosis (neutrophil extracellular traps/NETs release-associated pathogen-induced cell death), and entosis (cell-in-cell invasion)-associated cell death.

**Figure 8:**
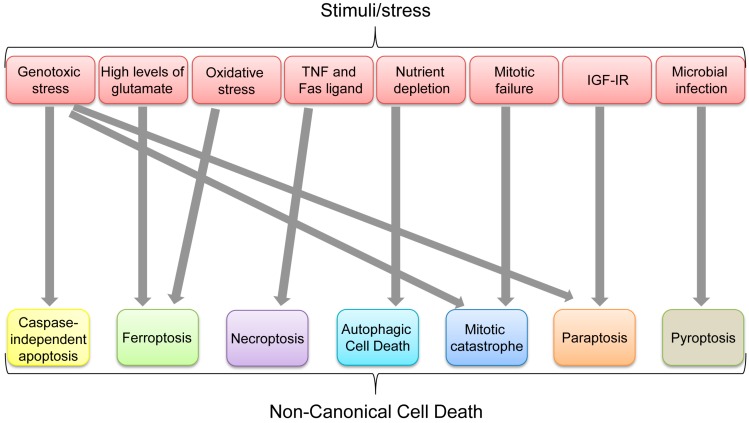
Non-canonical cell death induced by various stimuli/stress.

Aforementioned non-canonical cell death pathways can ultimately be used to get rid of unwanted cells, depending on physical, biochemical, pathological, chemical, or environmental conditions. Multiple types of cell death could be induced by certain stresses or stimuli. Indeed, some apoptosis-inducing stimuli, such as TNF, can also induce necroptosis when caspases are inhibited, while genotoxic stress can induce CIA, mitotic catastrophe, and paraptosis (Figure 8). However, each non-canonical cell death seems to have its favorable stimulus (Figure 8). It is also unclear how p53 preferentially selects its target genes involved in different cell death mechanisms and whether there are any dominant pathways or stimuli leading to a specific type of cell death in a p53-dependent or independent manner. In summary, p53 has critical roles in inducing each non-canonical cell death as listed in Table 1; however, it remains unclear whether p53 has any integrated roles in the whole processes of non-canonical cell death.

Importantly, since cancer cells are frequently deficient in the apoptotic pathway, capitalizing on the remaining non-canonical cell death mechanisms would be crucial for the development of novel anti-cancer therapies. Towards this goal, it is important to further our understanding of these cell death mechanisms and their relationship with p53.

## Figures and Tables

**Table 1:** p53 mediated non-canonical cell death pathways.

Mode of Cell Death	Definition	Major Mediators	Role of p53	Reference
Caspase-independent apoptosis (CIA)	Cell death occurring independently of caspases	AIF, EndoG	p53 transcriptionally upregulates AIF and also induces cytoplasmic translocation of AIF from mitochondria.	[33,34,35,45]
Ferroptosis	Iron-dependent regulated cell death	GPX4, SLC7A11	p53 transcriptionally represses SLC7A11.	[57]
Necroptosis (programmed necrotic cell death)	The regulated form of necrotic cell death	RIPK1/RIPK3, cypD, Cathepsin Q	p53 transactivates cathepsin Q, indirectly increases RIPK1/RIPK3 via the NRF-miR-873 axis, and directly interacts with cypD in mitochondria.	[64,67,68,73]
Autophagic cell death	Non-apoptotic, non-necrotic cell death resulting from the process of autophagy	ATG proteins, Beclin-1, DRAM	Nuclear p53 increases TSC2 and AMPK levels to inhibit mTOR activity, as well as increases DRAM levels, promoting the autophagic process. Cytoplasmic p53 binds with Beclin-1 and promotes its degradation to inhibit autophagy.	[87,88,89,91,92,93,98]
Mitotic catastrophe	Cell death caused by impaired mitosis	Cdk1	p53 inhibits transcription of cdk1.	[109,110,111]
Paraptosis	Programmed cell death induced by IGF-IR with swelling of mitochondria or endoplasmic reticulum (ER) and cytoplasmic vacuolization	IGF-IR, ALG-2-interacting protein (AIP1, also known as ALG-2 interacting protein-X, Alix)	Δ40p53 (p44) upregulates IGF-IR.	[135,139,141]
Pyroptosis	Inflammatory form of regulated cell death which is triggered by microbial infection	Caspase-1	p53 transactivates caspase-1, but its direct involvement in pyroptosis remains unclear.	[143,149]
Efferocytosis	The process by which phagocytes engulf and digest dead or dying cells	miR-34a	p53 transcriptionally upregulates miR-34a.	[155,156]
